# SARS-CoV-2 may play a direct role in the pathogenesis of posterior reversible encephalopathy syndrome (PRES) associated with COVID-19: A CARE-compliant case report and literature review

**DOI:** 10.1097/MD.0000000000037192

**Published:** 2023-02-02

**Authors:** Lishen Wang, Zhihan Wang, Rui Huang, Weishuai Li, Dongming Zheng

**Affiliations:** a Department of Neurology, Shengjing Hospital of China Medical University, Shenyang, China.

**Keywords:** COVID-19, endothelial dysfunction, infection, posterior reversible encephalopathy syndrome

## Abstract

**Rationale:**

During the past 3 years of the corona virus disease 2019 (COVID-19) pandemic, COVID-19 has been recognized to cause various neurological complications, including rare posterior reversible encephalopathy syndrome (PRES). In previously reported cases of PRES associated with COVID-19, the majority of patients had severe COVID-19 infection and known predisposing factors for PRES, such as uncontrolled hypertension, renal dysfunction, and use of immunosuppressants. It remains unclear whether these risk factors or infection with severe acute respiratory syndrome coronavirus 2 (SARS-CoV-2) contributes to the development of PRES in these patients. Here we report a special case of PRES associated with COVID-19 without any known risk factors for PRES, indicating the SARS-CoV-2’s direct role in the pathogenesis of PRES associated with COVID-19.

**Patient concerns:**

An 18-year-old female patient presented to the emergency department with abdominal pain. Preliminary investigations showed no abnormalities, except for positive results in novel coronavirus nucleic acid tests using oropharyngeal swabs. However, the patient subsequently developed tonic–clonic seizures, headaches, and vomiting on the second day. Extensive investigations have been performed, including brain MRI and lumbar puncture. Brain MRI showed hypointense T1-weighted and hyperintense T2-weighted lesions in the bilateral occipital, frontal, and parietal cortices without enhancement effect. Blood and cerebrospinal fluid analyses yielded negative results. The patient had no hypertension, renal insufficiency, autoimmune disease, or the use of immunosuppressants or cytotoxic drugs.

**Diagnoses:**

PRES was diagnosed based on the clinical features and typical MRI findings of PRES.

**Interventions:**

Symptomatic treatments such as anticonvulsants were administered to the patients.

**Outcomes:**

The patient fully recovered within 1 week. The initial MRI abnormalities also disappeared completely on a second MR examination performed 11 days later, supporting the diagnosis of PRES. The patient was followed up for 6 months and remained in a normal state.

**Lessons:**

The current case had no classical risk factors for PRES, indicating that although the cause of PRES in COVID-19 patients may be multifactorial, the infection of SARS-CoV-2 may play a direct role in the pathogenesis of PRES associated with COVID-19.

## 1. Introduction

During the COVID-19 pandemic over the past 3 years, clinicians have become aware that the infection is not limited to the respiratory system but may also involve other systems. The Neuro COVID-19 is of particular interest as many clinical subtypes under this umbrella term, such as encephalitis, encephalomyelitis, cerebral hemorrhage, and leukoencephalopathy, can have extremely severe consequences.^[1,2]^ In addition, rare neurological complications associated with COVID-19, such as posterior reversible encephalopathy syndrome (PRES),^[3,4]^ have been reported. PRES is a rare acute neurological syndrome with headache, consciousness disorders, seizures, visual disturbances, and focal neurological dysfunction as its primary clinical manifestations. Common precipitating factors include rapidly elevated blood pressure, eclampsia, renal insufficiency, autoimmune diseases, and the use of immunosuppressants or cytotoxic drugs. The dysfunction of cerebrovascular autoregulation caused by these factors via specific mechanisms is the most important theory regarding PRES occurrence.^[5]^

It remains unclear how COVID-19 leads to PRES. The vast majority of previous cases of PRES associated with COVID-19 occurred in patients with severe COVID-19 who received mechanical ventilation for hypoxemia, had poorly controlled hypertension, and were taking immunosuppressive medications.^[3]^ As all of these factors may be associated with the development of PRES, determining the core mechanism by which COVID-19 causes PRES in these patients is difficult. Here, we report a case of PRES in an 18-year-old female with mild severe acute respiratory syndrome coronavirus 2 (SARS-CoV-2) infection who had no hypertension, renal dysfunction, respiratory failure, or immunosuppressant use. We also conducted a literature search using PubMed for similar cases of COVID-19-associated PRES without known precipitating factors. We believe these cases help to support the more important role of the virus in the pathogenesis of PRES associated with COVID-19 than hypertension or the use of immunosuppressants.

## 2. Case presentation

**Figure 1.**
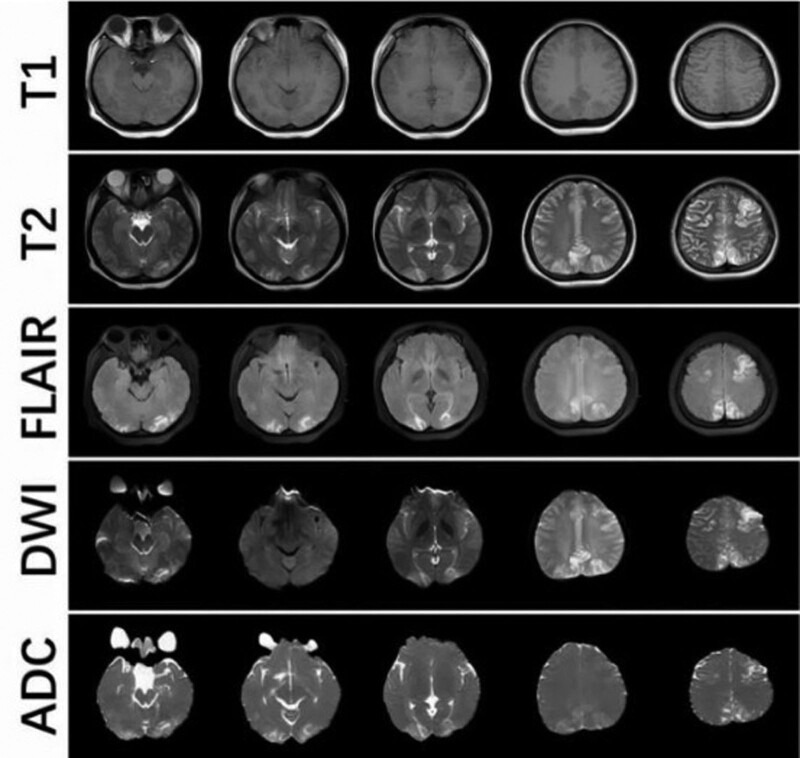
The patient’s first head MRI showed bilateral long T1-weighted and long T2-weighted signal lesions in the occipital, frontal, and parietal cortices, which were more evident on fluid-attenuated inversion recovery (FLAIR) sequences. On DWI and ADC maps. The lesions had slightly higher signal intensities. These lesions had no effect on the enhancement of scans (not included). ADC = apparent diffusion coefficient, DWI = diffusion weighted imaging, MRI = magnetic resonance imaging.

**Figure 2.**
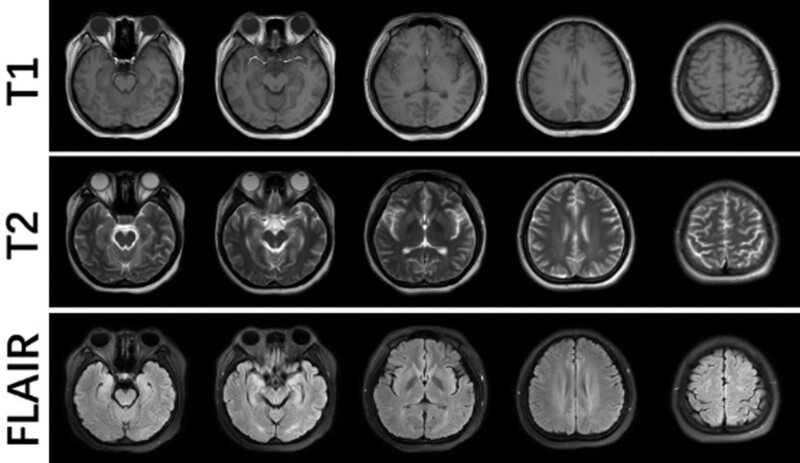
Eleven days after the first MRI scan, the second scan showed that the abnormal signal had completely disappeared. MRI = magnetic resonance imaging.

The patient is an 18-year-old woman who presented to our emergency department with the complaint of “abdominal pain for 2 days.” The abdominal pain was periumbilical and mild, with no nausea, vomiting, fever, or diarrhea. The patient had low-grade fever and pharyngeal pain for 2 days before presentation, with no cough or dyspnea, which had resolved without treatment. Her vital signs were as follows: blood pressure, 127/84 mm Hg, temperature 36.7°C, heart rate, 81 beats/min; and respiratory rate, 22 breaths/min. Routine blood and biochemical tests, chest computed tomography (CT), and a whole abdomen enhanced CT showed no abnormalities. However, 2 oropharyngeal swabs for novel coronavirus (nCoV) nucleic acid testing revealed a SARS-CoV-2 infection. On the second day in the emergency room, she experienced a sudden onset of convulsions, manifested by unconsciousness, a locked jaw, upturned eyes, and rigid limbs with shaking, which lasted for 2 minutes. After restoration of consciousness, the patient experienced headache with nausea and vomiting. A neurological examination revealed no positive findings. A CT scan of the head was performed immediately and showed no abnormalities. A lumbar puncture was performed on the same day with normal results: cerebrospinal fluid pressure of 140 mm H_2_O; white blood cell count of 3 × 10^6^/L; and protein, glucose, and chloride concentrations of 0.22 g/L, 4.21 mmol/L, and 120.6 mmol/L respectively. Later, she experienced several tonic–clonic seizures lasting 1 to 2 minutes each. Sodium valproate was administered intravenously to control convulsions. On day 3, a contrast-enhanced magnetic resonance imaging scan of the head revealed hypointense T1-weighted and hyperintense T2-weighted lesions in the bilateral occipital, frontal, and parietal cortices without enhancement effects (Fig. 1). These lesions showed a slightly higher signal change on diffusion-weighted imaging sequences and apparent diffusion coefficient maps. The patient was admitted to the neurology ward with a diagnosis of possible PRES based on the patient’s clinical presentation and imaging findings. The patient’s past medical records were unremarkable. She had no recent vaccinations, drugs, or toxic exposures. After admission, she showed no positive neurological signs during neurological examination. No further seizures occurred and the headache gradually subsided. Sodium valproate was administered orally at a dose of 500 mg twice daily. During hospitalization, serum complement (C3/C4), immunoglobulin (IgA, IgG, IgM), antinuclear antibody series, autoimmune small vasculitis-associated antibodies (ANCA, MPO, PR3), ASO, CRP, and RF levels were tested without abnormal findings. Another head magnetic resonance imaging scan was performed 11 days after the initial scan, which showed that the abnormal signals in the original scan had disappeared completely (Fig. 2). The patient was discharged without residual symptoms. The oral sodium valproate was continued for another 3 months before being stopped, and the patient did not experience any convulsions after discharge. She was followed up for 6 months and remained in a normal state.

This study was approved by the Ethics Committee of Shengjing Hospital of China Medical University, and was conducted in accordance with the Declaration of Helsinki. The patient provided written informed consent for participation in this study and for the publication of its findings.

## 3. Discussion

Kishfy et al^[6]^ first reported a case series of PRES associated with SARS-CoV-2 infection, and a gradual increase in similar case reports has led to the conclusion that PRES may be a neurological complication of COVID-19. However, PRES associated with COVID-19 remains an infrequent form of neurological complication, which includes stroke, seizures, meningitis, and encephalitis.^[2]^ The exact prevalence of PRES in COVID-19 patients remains unknown. A survey performed in 2020 with 278 COVID-19 patients who underwent brain imaging revealed a 1.1% prevalence of PRES.^[7]^ The clinical presentation of patients with PRES associated with COVID-19 is not significantly different from that of patients with other causes of PRES, which are also characterized by altered mental status, seizures, and visual disturbances and have typical PRES imaging changes.^[4]^ Overall, the majority of patients with COVID-19-associated PRES were patients with severe COVID-19,^[6,8]^ who suffered from severe hypoxemia requiring ventilatory support, uncontrolled hypertension, renal insufficiency, and the use of a variety of COVID-19 medications, such as immunosuppressive agents or hydroxychloroquine. As all of these factors can induce PRES, determining the main cause of COVID-19 leading to PRES is difficult.

Dysfunction in cerebrovascular autoregulation is a central mechanism in the development of PRES. Theoretically, impaired cerebrovascular autoregulation in PRES associated with COVID-19 could be related to the following factors: hypoxemia, an abrupt increase in blood pressure, angiotensin-converting enzyme 2 (ACE2) receptor-mediated endothelial dysfunction, vascular endothelial damage caused by the inflammation storm, and use of medications related to PRES. First, SARS-CoV-2 may be associated with severe pulmonary dysfunction due to inflammation and edema caused by viral proliferation in the lung tissue, which impairs alveolar gas exchange, leading to cerebrovascular hypoxia, anaerobic metabolism, and lactic acid accumulation, resulting in focal cerebrovascular diastolic and systolic dysfunction regulation and subsequent neurological damage.^[9]^ In case studies of PRES associated with COVID-19, more than half^[10,11]^ or even 100%^[3]^ of the patients received ventilator support for hypoxemia, indicating that hypoxemia plays a non-negligible role in PRES associated with COVID-19. Second, when blood pressure reaches the level of a hypertensive crisis or when large fluctuations occur in BP beyond the range of cerebrovascular autoregulation mechanisms, disruption of the blood–brain barrier causes cerebrovascular hyperperfusion and cerebrovascular leakage, leading to vasogenic edema, which is one of the most common mechanisms for the development of PRES.^[10,12]^ Extremely high blood has been reported in some patients with PRES associated with COVID-19, which may account for the onset of PRES in these patients. However, the proportion of hypertension in patients with COVID-19-associated PRES is not high, as shown in a Meta-analysis of Iftikhar et al,^[4]^ which revealed that only 28.6% of patients with COVID-19-associated PRES had hypertension. This indicates that hypertension may not be a major factor in the development of PRES caused by COVID-19. Third, SARS-CoV-2 enters human cells via the ACE2 receptor.^[13]^ ACE2 is widely expressed in humans, especially in the pulmonary epithelial cells, intestines, kidneys, hearts, and blood vessels.^[14]^ SARS-CoV-2 can downregulate ACE2 expression by directly binding to and attacking ACE2 receptors on endothelial cells, leading to excessive activation of the ACE/Ang II/AT1 axis and inhibition of the ACE2/Ang-(1-7)/MasR axis. This results in vascular pathological changes, such as increased permeability, inflammatory response, and oxidative stress, ultimately leading to damage to endothelial function,^[15,16]^ degradation of vascular endothelial junction proteins,^[17]^ and disruption of the blood–brain barrier.^[9]^ All of these changes can lead to endothelial inflammation and body fluid extravasation, resulting in PRES-associated vasogenic edema. Moreover, patients infected with SARS-CoV-2 have Th17/Treg cell dysfunction and immune hyperactivation, which results in the secretion of large amounts of proinflammatory cytokines.^[18]^ The binding of SARS-CoV-2 to ACE2 receptors can also stimulate the release of cytokines (ICAM-1, VCAM-1, and MCP-1) and proinflammatory molecules (IL-6, IL-8, and PAI-1), which can lead to cytokine release syndrome and damage the vascular endothelium.^[19–21]^ Finally, some patients with COVID-19-associated PRES have utilized medications, such as tocilizumab and hydroxychloroquine,^[22,23]^ which have been reported to induce PRES.^[24,25]^ More patients with COVID-19 have received steroid therapy, and although controversial, steroids have been suggested to be associated with the development of PRES.^[26]^

Similar to our case, Al Haboob^[27]^ and Suwanto and Ferrriastuti^[28]^ also reported pediatric cases of PRES associated with COVID-19 that had no evidence of hypertension or any risk factors for PRES. All these cases had no significant involvement of systems other than the central nervous system during SARS-CoV-2 infection. They hadn’t use any suspected medications related to PRES. These cases suggest that, among the aforementioned potential mechanisms, cerebrovascular impairment caused by SARS-CoV-2 may be a key factor in the development of PRES in these patients. We speculate that even in PRES patients with severe COVID-19, direct endothelial damage caused by SARS-CoV-2 should also be considered as a non-negligible precipitating factor of PRES. H1N1 influenza was known in humans long before COVID-19. It can cause severe pneumonia, leading to respiratory failure and trigger immune-inflammatory factor storms similar to SARS-CoV-2, resulting in many deaths each year. However, case reports on H1N1 influenza-induced PRES in PubMed are scarce. Studies on pulmonary vessels have shown that SARS-CoV-2 causes considerably more endothelial damage than H1N1.^[29,30]^ This may explain in part why COVID-19 causes more cases of PRES than H1N1 influenza, supporting the critical role of SARS-CoV-2 in the pathogenesis of PRES in COVID-19 patients.

There are several limitations of this report. Firstly, despite our comprehensive literature review that identified a few analogous cases, the sample size was too small for a meta-analysis. Thus the strength of our conclusions is limited. A significant shortfall in the existing case reports is the lack of detailed clinical information, such as baseline blood pressure data. This gap prevents a definite assessment of whether high blood pressure is a primary causative factor for PRES in these patients. If hypertension is present but the levels are close to normal, its connection with PRES onset may be coincidental rather than causal. If this is true, the number of patients in whom PRES is directly triggered by SARS-CoV-2 infection could be significantly higher than currently estimated. Second, our study did not include assays for COVID-19-associated cytokines, such as interleukins, IFN-γ, TNF-α, and etc. Assessing these biomarkers might offer crucial insights into the pathogenesis of PRES.

## 4. Conclusion

The cases in the present study shed new light on the understanding of the mechanisms underlying PRES associated with COVID-19. In general, current basic and clinical studies show that although the mechanism of PRES in COVID-19 patients may be multifactorial and complex, vascular endothelial dysfunction caused by SARS-CoV-2 infection may play a critical role in triggering PRES.

## Author contributions

**Conceptualization:** Dongming Zheng.

**Data curation:** Lishen Wang, Zhihan Wang.

**Funding acquisition:** Dongming Zheng.

**Project administration:** Rui Huang.

**Writing – original draft:** Lishen Wang.

**Writing – review & editing:** Weishuai Li, Dongming Zheng.
